# Introducing the National Library for Health Skin Conditions Specialist Library

**DOI:** 10.1186/1471-5945-5-4

**Published:** 2005-04-26

**Authors:** Douglas Grindlay, Maged N Kamel Boulos, Hywel C Williams

**Affiliations:** 1 National Library for Health Skin Conditions Specialist Library, Centre of Evidence-Based Dermatology, Queen's Medical Centre, University of Nottingham, Nottingham NG7 2UH, UK; 2 School for Health, University of Bath, Claverton Down, Bath BA2 7AY, UK

## Abstract

**Background:**

This paper introduces the new National Library for Health Skin Conditions Specialist Library .

**Description:**

The aims, scope and audience of the new NLH Skin Conditions Specialist Library, and the composition and functions of its core Project Team, Editorial Team and Stakeholders Group are described. The Library's collection building strategy, resource and information types, editorial policies, quality checklist, taxonomy for content indexing, organisation and navigation, and user interface are all presented in detail. The paper also explores the expected impact and utility of the new Library, as well as some possible future directions for further development.

**Conclusion:**

The Skin Conditions Specialist Library is not just another new Web site that dermatologists might want to add to their Internet favourites then forget about it. It is intended to be a practical, "one-stop shop" dermatology information service for everyday practical use, offering high quality, up-to-date resources, and adopting robust evidence-based and knowledge management approaches.

## Background

### Quality information all in one place

Dermatology is currently undergoing enormous changes in the way it is practised, and much of this is a result of a fundamental change in the manner in which information is exchanged through information technology and the Internet. The Web is increasingly becoming an indispensable tool for healthcare professionals caring for people with skin disease in their everyday clinical practice [1-8].

There are many quality online information resources on skin conditions, but locating these sources of information quickly and easily, and bringing together related information into one place, remains a big challenge for the average Internet user. Internet search engines such as Google  or Google Scholar  can be tried, but such an approach can be very time consuming and frustrating. Search engine results, including those from Google, contain too much "noise" in the form of irrelevant or low-quality material. Choosing suitable search terms, including skin disease or drug synonyms/variants, is an art in itself, and once a list of hits is obtained, the user has to wade through them to separate "the wheat from the chaff".

Although some quality dermatology Internet portals specialise in images, patient information leaflets, online tutorials, etc., nowhere does this information appear to be pulled together into one trustworthy source.

**Figure 1:**
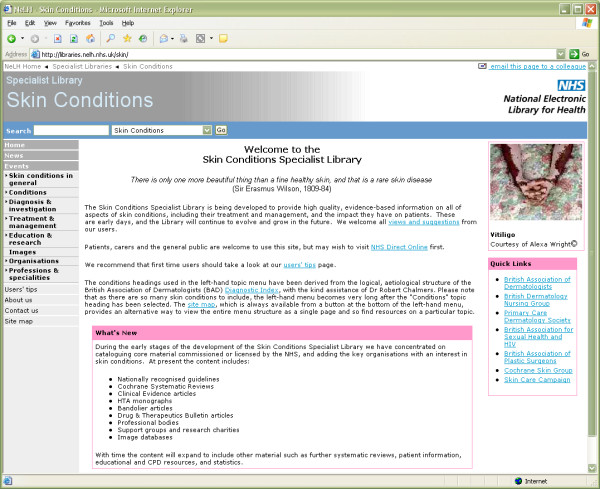
**Homepage of the NLH Skin Conditions Specialist Library**. Screenshot of the homepage of the NLH Skin Conditions Specialist Library  as launched in February 2005.

This is where the new UK National Library for Health Skin Conditions Specialist Library ( – Figure 1) should help. This new online digital library aims to catalogue, organise and harness the Web to the advantage of our users – primarily UK healthcare professionals caring for people with skin disease.

### How the Skin Conditions Specialist Library fits into the National Library for Health

In 2003, 19 Specialist Libraries were commissioned within the National electronic Library for Health (NeLH – ), to bring together resources for a range of clinical subject areas. Just as there are specialist sections in a real library branching off from the central general reading section, so too there are virtual specialist libraries in the NeLH, catering for more specialist groups and knowledge requirements. The Skin Conditions Specialist Library was one of these, and now even more Specialist Libraries are in the process of being commissioned, taking the number up to 26.

The aim of the NeLH was to organise clinical knowledge and to promote evidence-based decision making. The NeLH brought together over 70 electronic resources such as sources of guidelines, systematic reviews and bibliographic databases in one place, so that they could be accessed at any time wherever an Internet connection is available. Examples of key generic resources included in the NeLH are shown in Table 1.

**Table 1:** Examples of key generic resources included in the NLH. Examples of key generic resources (or core content) included in the National Library for Health . Access to all of these information sources in full is now completely free, although some, such as *Drug and Therapeutics Bulletin *and many of the full-text journals, require a valid Athens account.

**Resource**	**Internet address**
National Institute for Clinical Excellence (NICE) Guidelines	
NeLH Guidelines Finder	
PRODIGY	
Hitting the Headlines	
Cochrane Database of Systematic Reviews	
NHS Health Technology Assessment Programme	
CRD Database of Abstracts of Reviews of Effects (DARE)	
CRD Health Technology Assessment (HTA) Database	
Clinical Evidence	
Drug and Therapeutics Bulletin	
Bandolier	
Full-text journals	

Recently, the NeLH was subsumed into a bigger whole, the National Library for Health (NLH), with a new URL and home page . Hence, the Skin Conditions Specialist Library is now to be known as the NLH Skin Conditions Specialist Library. The NLH is a major component of the National Knowledge Service , which in turn is part of England's National Programme for IT . The NLH is funded by the NHS (National health Service) to combine the national digital resource managed by the existing NeLH with the physical content of books and journals and the skills and resources of health libraries (i.e. libraries in the NHS, health libraries in Higher Education, and the libraries of national organisations such as Royal Colleges and major charities).

## Construction and content

### Aims and scope

The NLH Skin Conditions Specialist Library  is an Internet information gateway for dermatology. It aims to provide an organised, easily accessible and up-to-date directory of key documents, reviewed evidence, and appraised information on skin conditions, including patient information resources. The emphasis is on quality and an evidence-based approach.

The subject remit of the Skin Conditions Specialist Library is the diagnosis, treatment, management and prevention of skin conditions, and the effect of skin conditions on quality of life. The scope is deliberately wide and includes disfigurement, skin cancer, skin surgery, wound care, sexually transmitted diseases that affect the skin, alternative and complementary treatments, consumer skin care, and cosmetic aspects such as skin ageing.

Identification of potential subject overlap is important, so that the various Specialist Libraries within the NLH can work together collaboratively to avoid unnecessary duplication of effort. The database used for the NLH Specialist Libraries allows cross-referencing where resources overlap and a synergistic, efficient use of resources.

The Skin Conditions Specialist Library has identified subject overlaps with cancer, child health, wound care, plastic surgery, sexually transmitted diseases, allergy, and alternative and complementary medicine. It has been proposed that plastic surgery and disfigurement should be housed within the Skin Conditions Specialist Library, at least for now.

### Audience

Although anyone connected to the Internet in the UK and elsewhere in the world, including patients with skin diseases and their relatives, is able to access the Skin Conditions Specialist Library, the Library's purpose is primarily to support UK healthcare professionals. As the Library's content develops, it is hoped that the Skin Conditions Specialist Library will become an important and well-used resource for all National Health Service (NHS) professionals dealing with skin conditions, including dermatology consultants and specialist registrars, junior doctors, dermatology nurses, general practitioners, practice nurses and health visitors. The Library should also prove useful to medical students and student nurses studying dermatology at undergraduate and postgraduate levels.

### Core Project Team, Editorial Team and Stakeholders Group

The National Library for Health Skin Conditions Specialist Library is based at the Centre of Evidence Based Dermatology at the University of Nottingham (which also hosts the Cochrane Skin Group – ). The core Project Team consists of the Clinical Lead, Professor Hywel Williams, Professor of Dermato-Epidemiology and Co-ordinating Editor of the Cochrane Skin Group, and the Information Specialist, Dr Douglas Grindlay.

A small Editorial Team, which includes members from the British Association of Dermatologists (BAD – ) and the British Dermatological Nursing Group (BDNG – ), along with other information scientists, provides advice on major issues of policy, content and quality (see ).

A wide-ranging Stakeholders Group has also been set up to ensure that the needs and views of all potential users are taken into account. The Stakeholders Group includes representatives from professional organisations such as the BAD, the BDNG, the Primary Care Dermatology Society , the British Association for Sexual Health and HIV , the British Association of Plastic Surgeons , and the Cochrane Skin Group . There are also representatives from an overarching body for patient support groups (the Skin Care Campaign – ) and five of the larger patient support groups. Another group of stakeholders included are health information providers, with representation from other NLH Specialist Libraries, NHS Direct Online , the OMNI database , the TRIP database , and the Chartered Institute of Library and Information Professionals (CILIP – ).

### Resource and information types

Table 2 lists the resource and information types that are included in the National Library for Health Skin Conditions Specialist Library, and those that are excluded from it under present policy.

**Table 2:** Skin Conditions Specialist Library resource and information types. Resource and information types that are included in the National Library for Health Skin Conditions Specialist Library, and those that are excluded from it.

**Resource and information types included in the Skin Conditions Specialist Library**
- Health policies and strategies
- Health initiatives
- National standards
- National and selected international guidelines and guidance
- Commissioning statements and service specifications
- Health Care Needs Assessment (HCNA)
- Pathways (from )
- Audit
- Skin disease statistics
- National Service Frameworks (NSFs) – when available
- Systematic reviews
- Sources appraising primary research
- Critically Appraised Topics (CATs)
- Clinical answering services (e.g. has two dermatology questions as at 31 January 2005)
- Selected research studies on skin conditions (as determined by the Editorial Team)
- Bibliographic databases
- Internet resource guides and gateways
- Special resources and tools (e.g. special collections of electronic journals, diagnostic aids, and dermatology images – )
- Education, teaching and continuing professional development resources
- Evaluated patient information
- Professional bodies and health organisations
- Patient support groups and societies
- News
- Events


**Resource and information types are excluded from the Skin Conditions Specialist Library**

- Research protocols or research in progress
- Cochrane protocols – the full systematic review will be added once it is published
- Product information provided by product manufacturer or supplier that has not been independently evaluated
- Web sites of commercial organisations (e.g. pharmaceutical companies, private hospitals)
- Personal Web sites
- Sites under construction (unless significant information is available to Editorial Team at the time of assessment)
- Information in a language other than English

### Collection building strategy

Individual Web resources on specific topics are discovered through systematic and regular trawling and searching of a core set of "sources of resources" that have been identified for this purpose:

- Sources for guidelines, pathways and policy documents, such as the NLH Guidelines Finder , PRODIGY  and BAD 

- Sources for systematic reviews and quality-assured synopses of evidence, such as the Cochrane Library 

- Sources appraising primary research, such as Bandolier 

- Sources for education and continuing professional development, such as BAD and BDNG

- Internet resource guides, such as Health on the Net Foundation HONselect  and OMNI 

- Web sites of professional bodies, skin research charities, support groups and other organisations concerned with skin conditions

- Sources of evaluated patient information/leaflets, such as BAD and PRODIGY

- Sources for news and events, e.g. NLH Hitting the Headlines and other news feeds and e-mail alerts

The relevant sections of each "source of resources" (in particular content lists and indexes) will be regularly visited and scanned for new documents, with the date of each search and the search strategy being recorded.

Internet search engines will also be regularly searched for relevant resources, helped by meta-search tools like Copernic . Again, the date of each search and query terms used will be recorded.

### Quality standards and selection criteria

Quality benchmarking and filtering of candidate resources is an issue of prime importance in ensuring that only those resources providing the *best current *knowledge and evidence are indexed in the National Library for Health Skin Conditions Specialist Library [9]. Resources will be selected if they meet all of the following criteria:

1. The resource falls under the subject scope for the Skin Conditions Specialist Library and is relevant to its intended users.

2. The resource and information it contains are of a type specified for inclusion in the Skin Conditions Specialist Library (see 'Resource and information types' above).

3. The resource has a stable enough presence on the Internet to be useful.

4. The resource is freely available on the Internet, or is accessible to NHS health professionals.

5. The resource is not strictly local in context.

6. Authorship of the resource can be ascertained and contact details are available.

7. Qualified individuals or groups take responsibility for the resource, coming from reputable organisations or having recognised expertise and authority in the field.

8. There is a process of refereeing of the clinical content by qualified health professionals or appropriate specialists.

9. The source of the content is verifiable and there is proper attribution.

10. Sources of information are clearly identified.

11. The information is current (e.g. *latest *version of guidelines) and kept up-to-date. Resources published more than ten years ago will not be included, unless they are judged to have special significance and value by the Editorial Team. Inclusion of resources will be reconsidered every year, and resources will be removed if out-of-date or superseded by others.

12. In the view of the Editorial Team, the information does not have any inaccuracies or bias.

13. For sites sponsored by commercial companies, in particular drug companies, for a site to be accepted, any sponsorship should be explicit and transparent, the content should be editorially independent, and there should be explicit steps to minimise bias or commercial selectivity.

It should be noted that the quality criteria apply to the resources as catalogued, not to any further links they may contain.

An important policy of the Skin Conditions Specialist Library is that resources on national NHS Web sites and those which are identified via the NLH core content are automatically eligible for inclusion, unless specific reasons for exclusion are identified by the Editorial Team.

Patient information resources present particular problems with assessing provenance and quality. The resource *must *be written, edited or reviewed by a qualified medical professional and there should be an explicit process of medical refereeing and regular review. Priority will be given to patient information with acceptable accreditation criteria, such as NHS Information Partners Web sites  or the Centre for Health Information Quality (CHIQ) TriangleMark scheme . Readability of patient information material [10] may perhaps be considered in a future revision of our quality benchmarking criteria.

A formal record will be kept of all resources that are rejected, with reasons. For example, we have recently come across a non-UK online dermatology image database that seemed a very good candidate for inclusion in our resource database, but was nevertheless rejected on quality grounds because it had no information as to provenance and the authority of the person producing it.

### Taxonomy for content indexing, organisation and navigation

**Figure 2:**
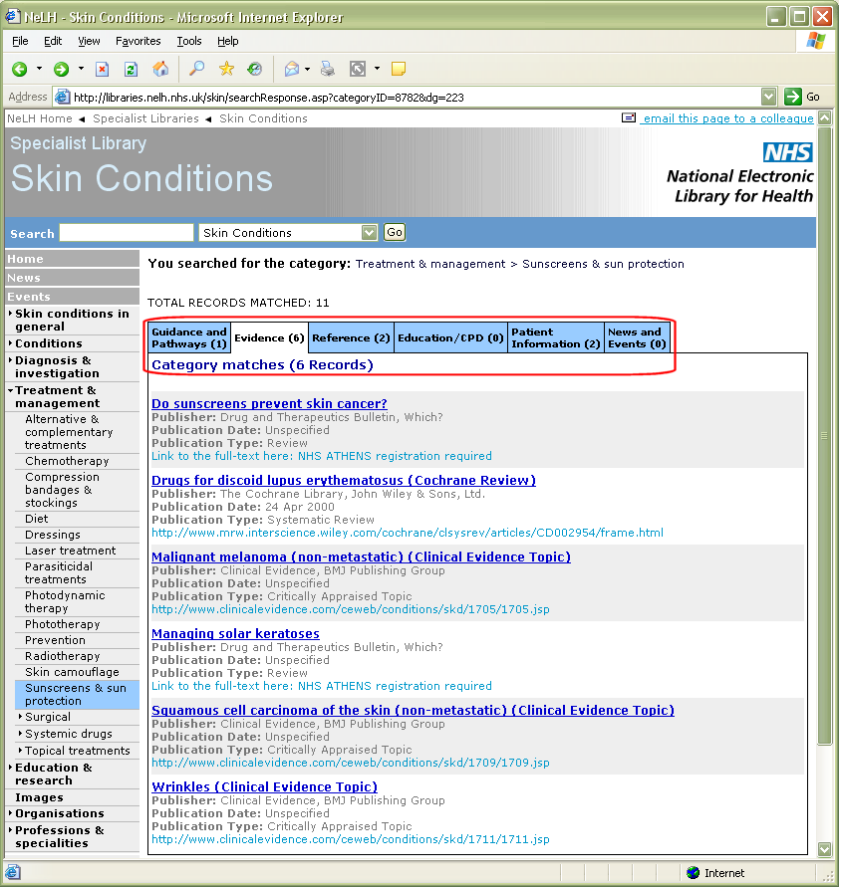
**Browsing the Library by skin topic and resource type**. Screenshot of a typical page from the National Library for Health Skin Conditions Specialist Library, for the menu topic "Treatment and management > Sunscreens & sun protection". On the left is the navigation menu which contains different topics such as skin conditions, treatments and organisations. Towards the top of the page, below the total number of records matched, can be seen the six tabs that are used to display records for different resource types. In this case the tab for "Evidence" has been selected, which includes systematic reviews.

**Figure 3:**
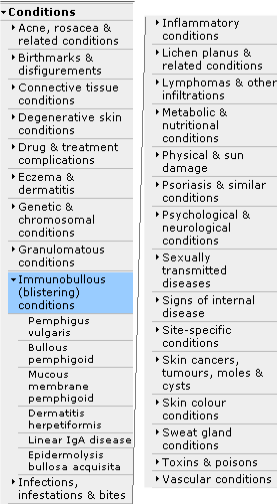
**The Library's navigation menu for skin conditions**. Screenshot of the Library's main topic headings for skin conditions, which are based on the BAD Diagnostic Index. The topic menu is always accessible on the left-hand side of the screen. The topic headings under 'Conditions' have been split into two in a graphics editor to reduce the height of this screenshot. Only higher-level categories are shown here, except for the 'Immunobullous (blistering) conditions' category (highlighted in light blue), which has been expanded.

A major advantage of the Skin Conditions Specialist Library is the organisation of its content by skin topic and resource type (e.g. Guidance and Pathways, Evidence, etc.), to facilitate retrieval by users (Figure 2). The navigation menu for skin conditions that is used has been derived from the logical, diagnostic structure of the BAD Diagnostic Index ( and Figure 3), with the kind assistance of Dr Robert Chalmers from the BAD Health Informatics sub-Committee, who is also a member of our Editorial Team. This means that the skin condition categories are mappable to those used by the Cochrane Skin Group. The structure of the BAD Index is due to be incorporated into the SNOMED CT (Systematized Nomenclature of Medicine Clinical Terms – ) terminology project in the future, so that as NHS electronic patient records are developed using SNOMED CT, it should be relatively easy to refer across to the Skin Conditions Specialist Library [11].

**Figure 4:**
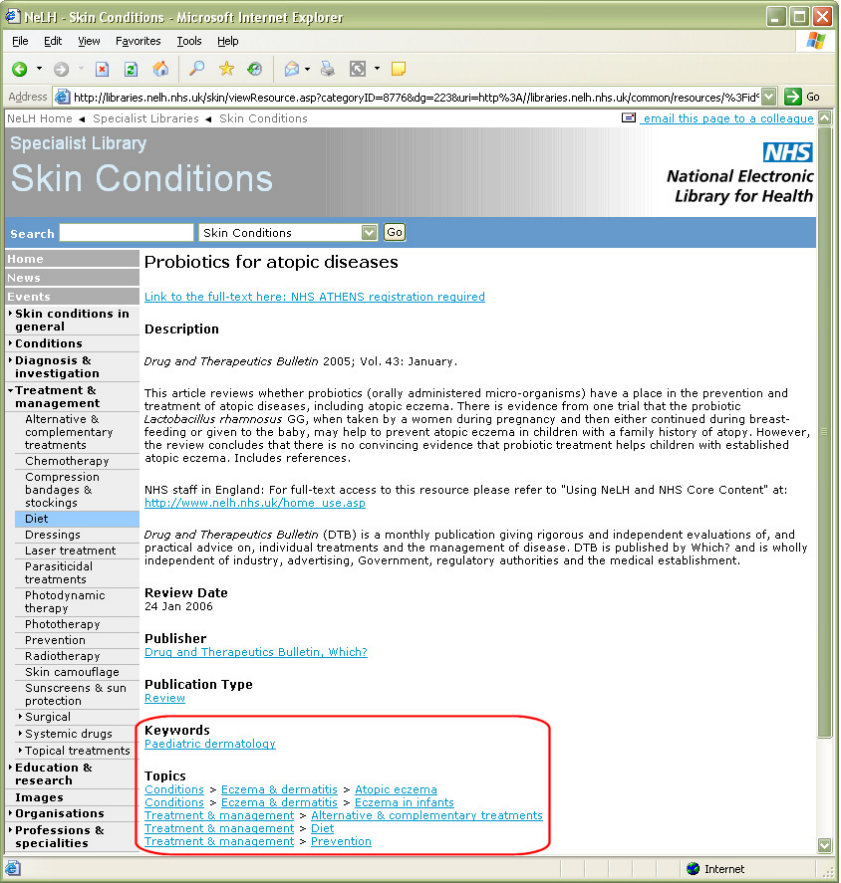
**A typical resource record from the new Skin Conditions Specialist Library**. Screenshot of a typical resource record page for a resource titled 'Probiotics for atopic diseases' from the new Skin Conditions Specialist Library database of resources. Note the 'Keywords' and 'Topics' fields near the bottom of the screenshot.

Tagging of resources in the Skin Conditions Specialist Library has been as detailed as possible, with tagging to multiple topics when this will help retrieval by users (Figure 4). Resources are tagged to the different Treatment & Management headings as well as to Condition headings. Thus, for example, it is possible to click on the menu topic for 'Methotrexate' (under 'Systemic drugs') and find all resources with significant discussion of this drug, such as guidelines, Cochrane Reviews, patient safety alerts, etc. Resources are also tagged for 'Speciality' when they are likely to be of interest to particular users, such as nursing (for pressure ulcers, leg ulcers, wounds, diabetic foot, etc.), surgery, or management.

However, the authors appreciate that their navigational classification of skin conditions based on the BAD Diagnostic Index will more likely suit the needs of specialist dermatologists rather than those of non-specialists and novices. In fact, in a classification task reported by Norman *et al*. [12], novices and non-specialists categorised skin lesions presented to them on slides by their surface features (e.g. scaly lesions), intermediates grouped the slides according to diagnosis, and expert dermatologists organised the slides according to superordinate categories such as viral infections, which reflected the underlying pathophysiological or aetiological taxonomy.

Fortunately, the NLH also incorporates powerful search functions, allowing users to search just the Skin Conditions Specialist Library, other Specialist Libraries, or all NLH collections in one go. This complementary search facility should care for the needs of many users, especially those who are non-specialists and less familiar with the formal classifications, terminology and diagnostic features of skin diseases used in our navigation menu.

### User interface and usability issues

The usability of the Skin Conditions Specialist Library is very much determined centrally by the parent organisation, the NLH. The user interface of the Library is consistent with the interfaces of other NLH Specialist Libraries in order to permit familiarity when moving from one Specialist Library to another, as a primary care practitioner will often need to do. In fact, nearly all of the Specialist Libraries are currently using the same content management system, and all should migrate to this system in the near future.

We know the fate of Web interfaces that fail to take into account all the stakeholders, and indeed the reader might think that in our case interface requirements are imposed by management rather than coming from actual users. However, this is not true for the NLH Specialist Libraries. Their shared content management system/interface, which is under continual improvement, is the result of ongoing analyses of users' requirements and consultations handled centrally by the NLH and covering a wide range of user types. One aim is to ensure interface consistency and a unified look and feel across the different parts of the NLH. Another aim is to free the time of the different Specialist Library Teams, so that they may concentrate on their primary roles in analysing the information needs of their specific users, and in finding and indexing quality online information.

## Utility and future developments

### A "one-stop-shop" service

The National Library for Health Skin Conditions Specialist Library is intended to provide a "one-stop shop", a single portal that can be used as a service to find quality, evidence-based information on dermatology that is relevant for UK health professionals. The Skin Conditions Specialist Library provides an organised, easily accessible and up-to-date collection of key documents, reviewed evidence, and appraised information on skin conditions, including selected patient information resources.

### Current priorities

As with the rest of the NLH, the initial focus on content is currently on NHS-branded or NHS-funded information. The Skin Conditions Specialist Library is very much a work in progress, and with time we expect it to grow to include more and more external resources (as long as they meet our quality standards – see 'Quality standards and selection criteria' above). The core resources will always be those based on the highest level of clinical evidence, such as systematic reviews, critically appraised synopses of the evidence and guidelines.

With just one full-time information specialist working on the Skin Conditions Specialist Library, the priority has to be searching for, identifying, and signposting existing information that is relevant and of high quality. The resources and opportunities for specific new content generation in house will be limited, at least initially.

### Patient information in the new Library

The initial source of information for patients is intended to be NHS Direct Online . However, there are so many chronic skin diseases where patients can become experts in their own conditions [13]. They will often want more information than can be obtained from typical consumer information sources such as NHS Direct Online, and they should be able to find this on the NLH.

For this reason, we hope the Library will also act as a single source for high quality patient information, such as that produced by the BAD, NHS Direct Online, DermNet , PRODIGY, and Informed Health Online . While this information will then be available directly to patients and relatives using the NLH, it will also be an important resource for dermatologists, GPs and nurses when they are looking for information to give out during consultations.

As skin conditions are often chronic, disfiguring and difficult to treat, we believe very strongly in this role for the Library and in the need for patients to be consulted as the Library develops (hence the inclusion of patient groups in our Stakeholders Group).

### Identifying gaps in evidence and knowledge base

The Skin Conditions Specialist Library should have an important role in identifying gaps in the evidence and knowledge base, which will help to prioritise future research, reviews of the evidence and policy development. Our consultations so far have identified a need for the Library to include quality, evidence-based information on the "rarer" skin conditions. Finding such information is probably the main reason experienced dermatologists will want to use the service initially, but ironically it is just this information that is most often lacking, at least in electronic form. As stated above, the main function of the Library is to bring together existing quality resources rather than generating new content. A potential role in discovering the gaps and fostering the production of appropriate electronic resources to fill them has been identified, working in co-operation with our Stakeholders' organisations.

### A current awareness service

Another important function of the Skin Conditions Specialist Library is to provide a current awareness service, to alert our users to important new research, systematic reviews, guidelines, policy developments, news and conferences that are relevant to the Library's remit. This function should develop and expand with time.

### Future directions

To be used regularly by its target audience, the Skin Conditions Specialist Library needs to provide an added-value service over and above a mere directory of links. The aim is to build an active community of repeat users who will make our Library their starting point in answering all their dermatology information needs.

Depending on users' particular needs and current situation, not all quality resources will have the same practical relevance to them (some *quality *resources might even act as distracting "noise"). One of the Library's major functions is knowledge management, i.e., avoiding information overload by striving to present results relevant to our users (according to their roles), and by highlighting the most important resources (e.g. those that provide a comprehensive overview of a subject). Key resources should eventually be tagged in the Library using a new "Editor's pick" function.

Another major issue for the Library to resolve is the tension between organising resources on the basis of a condition/diagnosis, and accessing information when a diagnosis is unknown, for example by symptoms/lesion morphology and location on the body. Both approaches are needed, but the latter would be important for non-specialists such as GPs and NHS Direct nurses, particularly in view of the large number of skin conditions that exist. Probably some form of diagnostic and learning facility/decision support tool will be required in the future. An example of such a tool is *VisualDX *. As well as helping to access information in the absence of a pre-existing diagnosis, a system of this kind would be invaluable for differential diagnosis and continuing professional development ("learn as you practise"). It would also be of great use to students. There are two (complementary) possibilities for such systems, either as a separate diagnostic program or as pop-up tool embedded into electronic patient records (contextual decision-support system). In the meantime, the Library already includes a topic heading for Images, with links to several dermatology image databases.

Another long-term goal for the Skin Conditions Specialist Library team is to develop special features such as learning and continuing professional development resources, quizzes and commissioned briefings. Contributions from users could also be incorporated, for example suggestions on useful Web sites, hence the importance of including a comment return system on the Library's homepage . Another possibility is to have a "Web site of the month" feature.

There is also the possibility of using RSS (Really Simple Syndication) feeds to provide our users with regular updates on relevant new Library content, a feature already available elsewhere on the NLH (see ).

These possibilities will be investigated further once the core Library content has been fully established.

## Conclusion

The new National Library for Health Skin Conditions Specialist Library is an exciting project that aspires to be of real, practical use to all health professionals who treat, manage and support people with skin conditions. The Library is not just another dermatology Web site that users might add to their Internet favourites, but rather a single source where users can view quality-assured information rapidly, easily and for free. It also has the potential to highlight existing gaps in the evidence and knowledge base for dermatology, and to foster the development of teaching and learning resources.

Although commissioned for, and targeted at, UK users, we hope that the free access to the Skin Conditions Specialist Library will mean that users from other countries around the world will visit and use our site.

We consider all users (and potential users) of the Skin Conditions Specialist Library as our stakeholders. Comments and suggestions for improvement are always welcome, and we hope with time to develop a community of interest in skin conditions in the UK supported by newsletters and regular updates via the Library pages.

## Competing interests

The author(s) declare that they have no competing interests.

## Authors' contributions

As member of the Library's Editorial Team, MNKB advised on and participated in formulating many of the Library's aspects, including its editorial policies and quality checklist. MNKB also conceived and drafted this manuscript. The Library's core Project Team comprises DG, the Library's Information Specialist, and HCW, the Library's Clinical Lead, who together are responsible for developing and managing the Library, and organising the activities of its Editorial Team and Stakeholders Group. DG and HCW also revised and edited the first drafts of this manuscript and provided valuable input that helped improving it. All authors read and approved the final manuscript.

## Pre-publication history

The pre-publication history for this paper can be accessed here:


